# Thin ceramic PZT dual- and multi-frequency pMUT arrays for photoacoustic imaging

**DOI:** 10.1038/s41378-022-00449-0

**Published:** 2022-11-17

**Authors:** Qincheng Zheng, Haoran Wang, Hao Yang, Huabei Jiang, Zhenfang Chen, Yao Lu, Philip X.-L. Feng, Huikai Xie

**Affiliations:** 1 School of Integrated Circuits and Electronics, Beijing Institute of Technology (BIT), 100081 Beijing, China; 2 Department of Electrical and Computer Engineering, University of Florida, Gainesville, FL 32611 USA; 3 Department of Medical Engineering, University of South Florida, Tampa, FL 33620 USA; 4 MEMS Engineering and Materials Inc., Sunnyvale, CA 94086 USA; 5 BIT Chongqing Institute of Microelectronics and Microsystems, 400030 Chongqing, China

**Keywords:** Sensors, NEMS

## Abstract

Miniaturized ultrasonic transducer arrays with multiple frequencies are key components in endoscopic photoacoustic imaging (PAI) systems to achieve high spatial resolution and large imaging depth for biomedical applications. In this article, we report on the development of ceramic thin-film PZT-based dual- and multi-frequency piezoelectric micromachined ultrasonic transducer (pMUT) arrays and the demonstration of their PAI applications. With chips sized 3.5 mm in length or 10 mm in diameter, square and ring-shaped pMUT arrays incorporating as many as 2520 pMUT elements and multiple frequencies ranging from 1 MHz to 8 MHz were developed for endoscopic PAI applications. Thin ceramic PZT with a thickness of 9 μm was obtained by wafer bonding and chemical mechanical polishing (CMP) techniques and employed as the piezoelectric layer of the pMUT arrays, whose piezoelectric constant *d*_31_ was measured to be as high as 140 pm/V. Benefiting from this high piezoelectric constant, the fabricated pMUT arrays exhibited high electromechanical coupling coefficients and large vibration displacements. In addition to electrical, mechanical, and acoustic characterization, PAI experiments with pencil leads embedded into an agar phantom were conducted with the fabricated dual- and multi-frequency pMUT arrays. Photoacoustic signals were successfully detected by pMUT elements with different frequencies and used to reconstruct single and fused photoacoustic images, which clearly demonstrated the advantages of using dual- and multi-frequency pMUT arrays to provide comprehensive photoacoustic images with high spatial resolution and large signal-to-noise ratio simultaneously.

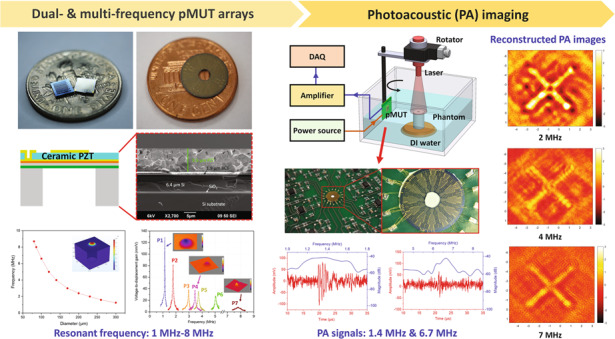

## Introduction

Photoacoustic imaging (PAI), with the advantages of rich optical contrast, deep penetration depth and high acoustic resolution, has been studied for decades and demonstrated for a wide variety of applications, such as breast cancer detection^1,2^, brain functional imaging^3^, human extremity imaging^4^, and hemodynamic studies^5,6^. In addition to benchtop PAI systems, endoscopic PAI has been extensively studied to diagnose diseases of internal organs and to provide accurate local information of deeply located tissues for surgical guidance^7,8^. For example, Jansen et al. demonstrated intravascular PAI of human coronary atherosclerotic plaque based on a 1.25-mm-diameter imaging catheter in 2011^9^. Xi et al. presented a microelectromechanical system (MEMS)-based intraoperative PAI probe and demonstrated its ability to evaluate breast tumor margins and inspect the resection of tumors during surgery in 2012^10^. Yang et al. reported a catheter-based photoacoustic endoscope that can be used for human gastrointestinal tract imaging applications in 2014^11^. Basij et al. presented a miniaturized phased-array ultrasound and photoacoustic endoscopic imaging system for cervical cancer diagnosis in 2019^12^. However, the development of endoscopic PAI is still in an early stage, with no mature photoacoustic endoscopes recognized in clinical settings. The main challenges include the integration of a light source, scanning components, and miniaturization of the imaging components while maintaining high performance.

In PAI, short laser pulses, usually on the order of nanoseconds, are used to illuminate and excite tissues to generate ultrasound signals with a broad bandwidth based on the photoacoustic effect^13^. The ultrasound signals propagate and are detected by a single element ultrasonic transducer (UT) or an array of UTs. UTs or UT arrays are key components in PAI systems, as their sensitivity, frequency, and bandwidth directly affect the signal-to-noise ratio (SNR), penetration depth, and imaging resolution of the PAI system. Higher-frequency UTs can achieve higher spatial resolutions, but the imaging depth is sacrificed due to higher acoustic attenuation and lower SNR^14^. Moreover, the selection of the center frequency of the UTs is dependent on the imaging targets since the frequency components of photoacoustic signals are related to the size of the absorber. Typically, smaller imaging targets exhibit higher-frequency photoacoustic signals^15^. Therefore, dual- and multi-frequency UTs or UT arrays are required for endoscopic PAI to visualize targets with multiple length scales and to simultaneously achieve high spatial resolution and large imaging depth.

However, conventional UTs working on the thickness extension mode of piezoelectric materials are expensive to be fabricated into arrays with multiple resonant frequencies since their resonant frequencies are directly determined by the thickness of the employed piezoelectric plate^16^. Their bulky size also makes it challenging to apply them in endoscopic PAI. Facilitated by MEMS technologies, micromachined ultrasonic transducers (MUTs), with the advantages of very small footprints and easy array fabrication at low cost, have been developed for endoscopic PAI applications, including capacitive MUTs (cMUTs) and piezoelectric MUTs (pMUTs)^17^. Multi-frequency cMUT arrays have been demonstrated for PAI with abilities to accurately reveal multiscale structures and to provide comprehensive images with high resolution and high contrast^18,19^. However, the high bias voltage and small capacitive gap requirements of cMUTs impose challenges for their biomedical application and fabrication processes. Their small capacitances also make them susceptible to parasitic effects and therefore require complex application-specified integrated circuits (ASICs). In contrast, pMUTs, with the advantages of easy fabrication, robustness against parasitic effects, and better design flexibility, are drawing increasing attention for endoscopic PAI applications^20,21^. Sputtered aluminum nitride (AlN), sol-gel lead zirconate titanate (PZT), and thin-film ceramic PZT have been employed to fabricate pMUTs working on the flexural vibration mode for PAI applications. For example, in 2013, Chen et al. presented a 2.89 MHz pMUT based on a sputtered AlN film and successfully obtained photoacoustic images of a human hair embedded in a phantom using the fabricated pMUT^22^. In 2018 and 2019, Dangi et al. reported pMUT arrays with resonant frequencies of 6–7 MHz based on a 0.65 µm-thick sol-gel PZT film and a 0.7-µm-thick sputtered AlN film, respectively, and demonstrated their PAI abilities and potential for integration into miniaturized probes^23,24^. In 2020, Wang et al. presented a 1.2 MHz pMUT array based on a 4 µm-thick ceramic PZT film with high piezoelectric constants for endoscopic PAI applications^25^. There have also been a few demonstrations of handheld or endoscopic PAI with pMUTs working on the thickness extension mode^11,26,27^. For example, in 2019, Liu et al. presented a handheld photoacoustic imager based on a 15 × 80 × 3 mm^3^ 2D pMUT array working at 2.25 MHz^28^. In 2015, Li et al. reported a catheter of 0.9 mm in diameter for intravascular PAI, where a single element pMUT with a dimension of 0.6 × 0.5 × 0.2 mm^3^ and a center frequency of 40 MHz was employed^29^. In terms of state-of-the-art pMUTs, the thickness extension mode pMUTs are typically fabricated based on ceramic PZT, single-crystal lead magnesium niobate-lead titanate (PMN-PT), and single-crystal lithium niobate (LiNbO_3_)^17^. Most thickness mode pMUTs applied in endoscopic PAI applications are single frequency and single element pMUTs because it is challenging for thickness mode pMUTs to be fabricated into arrays and operating at multiple frequencies^30,31^. In contrast, flexural vibration mode pMUTs are easier to be fabricated into large arrays. Typical piezoelectric thin films, such as PZT, AlN, and transferred LiNbO_3_ thin films, have been applied to fabricate flexural vibration mode pMUTs^32–34^. However, their applications in endoscopic PAI are still in an early stage, facing the challenges of limited film thickness and insufficient piezoelectric response. There is a lack of studies on dual- and multi-frequency pMUT arrays for PAI applications, and very limited PAI results with pMUT arrays have been shown.

In previous works, we proposed a 16 × 16 square-shaped dual-frequency pMUT array based on a 4-µm-thick ceramic PZT film operating at 1.2 MHz and 3.4 MHz for endoscopic PAI applications^35^. To increase the SNR and to obtain photoacoustic images with higher resolution and higher contrast, the sensing aperture of pMUT elements and the frequency range of pMUT arrays need to be further increased. Therefore, we present dual- and multi-frequency pMUT arrays based on a 9-µm-thick ceramic PZT film with resonant frequencies ranging from 1 MHz to 8 MHz for endoscopic PAI applications in this work. Some preliminary results based on square-shaped multi-frequency pMUT arrays were reported in ref. ^36^. This article presents the full design, fabrication, characterization, and PAI results of pMUT arrays in detail. In addition to the square-shaped multi-frequency pMUT array, a new, circular ring-shaped dual-frequency pMUT array is also developed to incorporate more pMUT elements and enable convenient light delivery for endoscopic PAI applications.

### Design concept and fabrication process

**Fig. 1:**
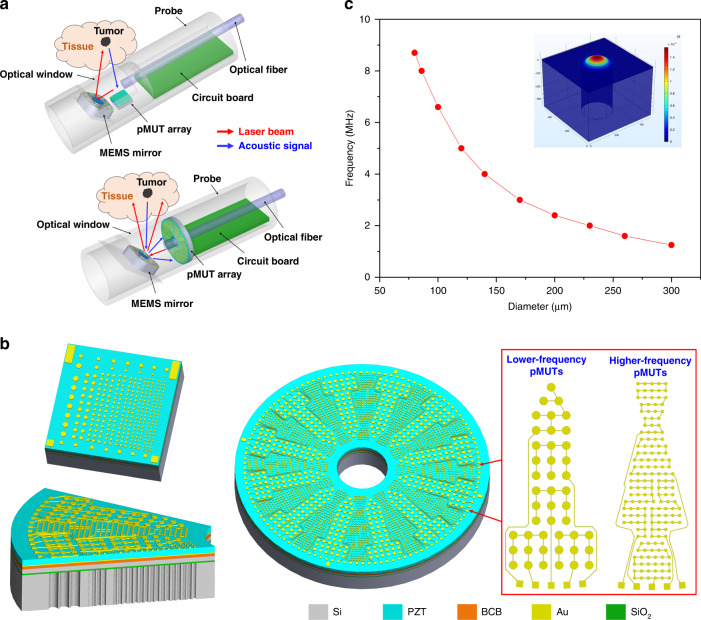
Dual- and multi-frequency pMUT array design. **a** Conceptual illustration of endoscopic PAI probes based on multi-frequency pMUT arrays (top) and dual-frequency pMUT arrays (bottom). **b** 3D models of the designed multi-frequency pMUT array (top left), dual-frequency pMUT array (right), and a part of the dual-frequency pMUT array in cross-sectional view (bottom left). **c** Simulated resonant frequencies of pMUTs with different diameters

Figure 1a illustrates the conceptual design of endoscopic PAI probes for square-shaped and ring-shaped pMUT arrays, where an optical fiber, a MEMS scanning mirror, and a pMUT array with a circuit board are assembled into the probe. A laser beam is delivered by an optical fiber and steered by the MEMS mirror, while laser-induced acoustic signals are detected by the pMUT array and processed by the circuit board. If the MEMS mirror performs one-dimensional (1D) angular scanning, 2D slices of cross-sectional photoacoustic images can be obtained. To conduct 3D imaging, the MEMS mirror must rotate in two transverse axes.

In this work, a square-shaped pMUT array with seven different resonant frequencies is designed to validate the PAI performance of multi-frequency pMUT arrays, while a ring-shaped dual-frequency pMUT array with better geometrical symmetry and more sensing elements is designed to improve imaging performance. Moreover, the central hole of the ring-shaped pMUT array enables the insertion of optical fibers for convenient light delivery in endoscopic PAI probes. Note that the MEMS mirror has not yet been implemented in the PAI system established in this work.

In endoscopic PAI probes, pMUTs work as ultrasound receivers. When photoacoustic waves in ultrasonic frequencies approach and strike the pMUT diaphragms, the diaphragms vibrate and generate electrical signals due to the direct piezoelectric effect. Figure 1b shows 3D models of the designed square-shaped multi-frequency pMUT array and ring-shaped dual-frequency pMUT array. Both consist of circular pMUT diaphragms suspended over acoustic cavities, which work at the flexural vibration with electrical charges generated by the *d*_31_ mode excitation of the embedded piezoelectric layers. The piezoelectric layer is a key component of pMUTs, as its piezoelectric properties directly affect the responsivities of the fabricated pMUTs. In this work, thin ceramic PZT obtained by wafer bonding and chemical mechanical polishing (CMP) techniques is employed as the piezoelectric layer because its piezoelectric coefficient is much greater than those of commonly used sputtered AlN and sol-gel PZT thin films^37,38^. Moreover, the use of thin ceramic PZT can achieve a lower processing temperature and a wider range of film thicknesses than the use of deposited AlN or PZT thin films, yielding films with lower stresses and facilitating the design of larger pMUTs with high resonance frequencies^39^. Ceramic PZT has been thinned to thicknesses as small as 4–5 µm for fabricating pMUTs and MEMS loud speakers, with excellent performance demonstrated^35,40^. To fabricate pMUTs with higher resonant frequencies and larger sensing apertures, the thickness of ceramic PZT is increased to 9 µm in this work. The obtained thin ceramic PZT layer is permanently bonded on a silicon-on-insulator (SOI) substrate using a thin layer of benzocyclobutene (BCB) polymer. A part of the ring-shaped pMUT array with a cross-sectional view is also shown in Fig. 1b, where each pMUT diaphragm is a multilayer structure composed of Au (350 nm)/PZT (9 µm)/Au (100 nm)/BCB (2 µm)/Si (6 µm)/SiO_2_ (1 µm) from the top to the bottom, with the thickness of each layer given in the corresponding parenthesis.

Depending on the flexural rigidity and the diameter-to-thickness ratio, the pMUT diaphragm can be modeled as a clamped heterostructure vibrating circular plate (disk) or membrane. Therefore, its fundamental flexural mode resonant frequency is determined by the material properties, residual stress, thickness, and diameter of the pMUT diaphragm. For the same multilayer stack, by tuning the diameter of the pMUT diaphragm, various resonant frequencies can be obtained and designed in one pMUT array. COMSOL Multiphysics software (COMSOL Inc., Burlington, USA) is used to simulate the fundamental resonant frequencies of pMUTs with different diameters, and the results are shown in Fig. 1c. Seven different resonant frequencies ranging from 1 MHz to 8 MHz have been selected with the corresponding pMUT elements designed in the multi-frequency pMUT array. Table 1 lists the designed diameters and resonant frequencies of the pMUT elements in the multi-frequency pMUT array. For each frequency design, the top electrodes of several pMUT elements are connected together as one channel to increase the sensing area. Individual pMUT elements are also designed as testing elements. There are 285 pMUT elements in total in the square-shaped pMUT array, and all of them share the same bottom electrode, with vias opened at the four corners of the chip, as shown in Fig. 1b.

**Table 1:** Designed diameters and resonant frequencies of the pMUT elements in the multi-frequency pMUT array

Element ID	P1	P2	P3	P4	P5	P6	P7
Diameter (µm)	300	230	170	152	140	120	86
Frequency (MHz)	1.2	2	3	3.5	4	5	8

To enable the integration of fiber optics and to achieve multichannel data acquisition in parallel at different locations, a ring-shaped dual-frequency pMUT array is also designed based on the same multilayer stack. There are 120 channels including 60 lower-frequency channels and 60 higher-frequency channels designed in the array. Thus, photoacoustic signals at different locations can be detected simultaneously for fast imaging without mechanically scanning transducers. As shown in Fig. 1b, the pMUT array can be divided into 12 sections by rotating at steps of 30° to form the full ring. There are 12 lower-frequency sections and 12 higher-frequency sections interlaced one by one, with each section including 5 channels along the radial direction. By integrating the pMUT elements labeled P2 and P7 in Table 1, resonant frequencies of 2 MHz and 8 MHz with a wide frequency separation are targeted to achieve high resolution and large imaging depth simultaneously in PAI. To increase the sensing aperture, each channel consists of multiple pMUT elements with their top electrodes connected. The inset of Fig. 1b shows a lower-frequency section and a higher-frequency section, each of which consists of 5 channels with multiple connected pMUT elements. To realize a high filling factor, each lower-frequency channel includes 9 pMUT elements, while each higher-frequency channel includes 33 pMUT elements due to the smaller size of the higher-frequency pMUTs. Therefore, 2520 pMUT elements are incorporated in an array, and 120 wire bonding pads connected to their top electrodes are arranged at the edge of the chip, forming a full circle. All of the pMUT elements share the same bottom electrode, with four vias opened on the chip.

**Fig. 2:**
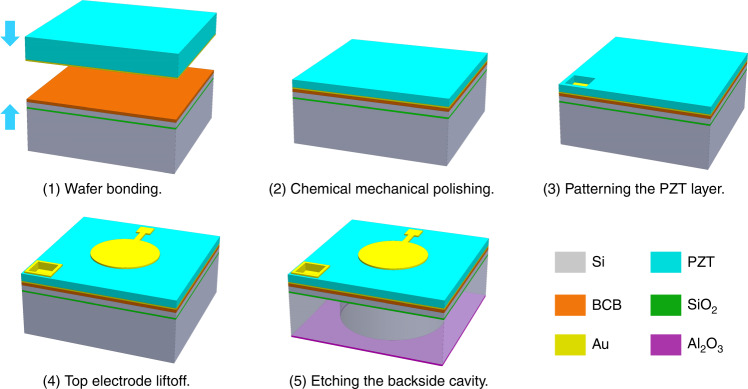
Illustration of the pMUT fabrication process flow. (1) wafer bonding, (2) chemical mechanical polishing, (3) patterning the PZT layer, (4) top electrode liftoff, and (5) etching the backside cavity

The designed dual- and multi-frequency pMUT arrays were fabricated from an SOI wafer and an off-shelf ceramic PZT wafer (PZT-5H, *d*_31_ = 300 pC/N, CTS Inc., USA), as illustrated in Fig. 2. The critical step in pMUT fabrication is the thinning and integration of the ceramic PZT wafer, which is achieved by wafer bonding and CMP techniques. First, the ceramic PZT wafer, with an initial thickness of 500 µm, is polished to have a smooth surface for sputtering Au as the bottom electrode. Subsequently, BCB is coated on an SOI wafer as the adhesion layer, and the metalized ceramic PZT wafer is permanently bonded with the SOI wafer at a temperature of 200 °C, which is well below the Curie temperature (242 °C) of PZT-5H (Fig. 2(1)). Then, the ceramic PZT is thinned to the designed thickness by a carefully tuned CMP process (Fig. 2(2)). Next, the PZT layer is patterned by photolithography #1 and wet etching using diluted fluoroboric acid (HBF_4_) to expose the bottom electrode^41^ (Fig. 2(3)). After that, the top electrode pattern is defined by photolithography #2 and formed by Au sputtering and liftoff process (Fig. 2(4)). Finally, the acoustic cavities of the pMUTs are fabricated by deep reactive ion etching (DRIE) of the silicon substrate from the backside with an Al_2_O_3_ layer as the hard mask (Fig. 2(5)). The Al_2_O_3_ mask is deposited by sputtering and patterned by two-side photolithography #3 and wet etching with diluted hydrofluoric acid.

**Fig. 3:**
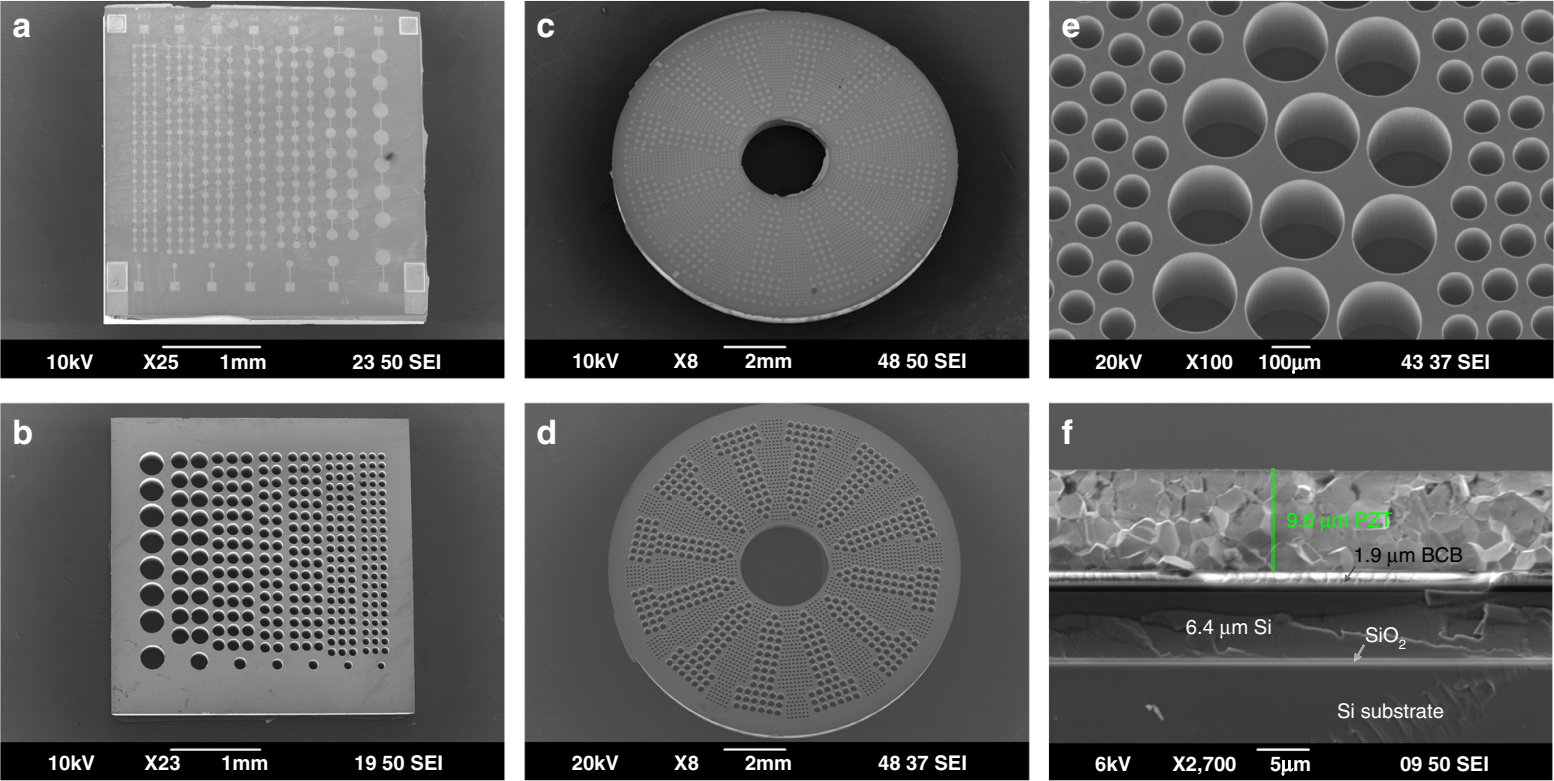
SEM images of the fabricated devices. **a** The front side and **b** back side of the fabricated multi-frequency pMUT array. **c** The front side, **d** the back side, and **e** sidewalls of the fabricated dual-frequency pMUT array. **f** A cross-sectional view of the multilayer stack

Figure 3 shows scanning electron microscopy (SEM) images of the fabricated multi-frequency and dual-frequency pMUT arrays. The square-shaped multi-frequency pMUT array has a chip size of 3.5 mm by 3.5 mm, and the corresponding front-view and backside-view SEM images are shown in Fig. 3a, b, respectively. The ring-shaped dual-frequency pMUT array has an outer diameter of 10 mm and an inner diameter of 2.5 mm, and the corresponding front-view and backside-view SEM images are shown in Fig. 3c, d, respectively. With careful control of the temperature and the etching and passivation cycles in the DRIE process, dense cavities with vertical sidewalls are obtained, as shown in Fig. 3e. Figure 3f shows a cross-sectional view of the fabricated layer stack, where the measured thickness of the ceramic PZT layer is ~9.6 μm. Measurements across the wafer show that the thickness of the ceramic PZT layer varies between 8 μm and 10 μm.

## Results and discussion

To evaluate the piezoelectric properties of the obtained thin ceramic PZT film and the performance of the fabricated pMUT arrays, various experiments in terms of electrical, mechanical, acoustical, and optical characterization were conducted. In the following, the measurement of the piezoelectric coefficient and the device performance are presented and discussed.

### Piezoelectric coefficient of thin-film ceramic PZT

**Fig. 4:**
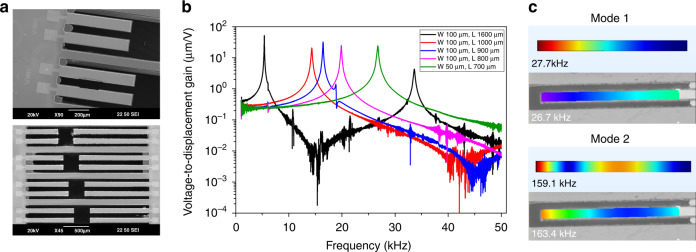
Characterization results of the fabricated piezoelectric actuators. **a** The zoom-in (top) and zoom-out (bottom) SEM images of the fabricated piezoelectric actuators. **b** Measured vibration frequency response. **c** Simulated and measured vibration mode shapes

To evaluate the *d*_31_ piezoelectric coefficient of the thin ceramic PZT film employed in this work, piezoelectric cantilever beams with different lengths (*L*) and widths (*W*) were designed based on the same layer stack of pMUT arrays and fabricated from the same wafer as the testing structures. SEM images of the fabricated piezoelectric cantilevers are shown in Fig. 4a, where the cantilever beams have the same width of 100 μm but different lengths ranging from 400 μm to 1600 μm.

Due to the converse piezoelectric effect, a lateral strain is generated and bends the piezoelectric cantilever actuator when a voltage is applied on the piezoelectric layer along its thickness direction. Combining the displacement measurement with the multimorph model of piezoelectric cantilever structures is an effective and accurate approach to derive the *d*_31_ coefficient of piezoelectric thin films^42^. In this work, the deflections of five piezoelectric actuators with different lengths and widths were measured by a laser Doppler vibrometer (LDV). The piezoelectric actuators were driven by a chirp signal with an amplitude of 0.1 *V*_rms_ and sweeping frequencies, while the displacements at the tip of the actuators were measured and recorded. The voltage-to-displacement gain in the frequency domain was calculated and shown in Fig. 4b, where different resonant frequencies are observed for the actuators with different dimensions. By defining multiple points on each actuator and scanning them one by one, the vibration mode shapes were measured and visualized. The resonant frequencies and vibration mode shapes were also simulated in COMSOL Multiphysics software, using the same dimensions as those of the measured piezoelectric actuators. Figure 4c shows the first two vibration mode shapes of a piezoelectric actuator (50 μm width, 700 μm length), where the simulation (top figures) and measurement (bottom figures) agree well.

With the measured voltage-to-displacement gain, the piezoelectric coefficient *d*_31_ is derived from equations given in^42^, which varies between 110 pm/V and 140 pm/V. In the calculation, the voltage-to-displacement gain at 1 kHz was used, which was well below the resonant frequency of the device. Within a certain error range caused by the layer thicknesses, Young’s moduli, and the assumption of a constant radius of curvature, the result validates the superior piezoelectric properties of thin ceramic PZT. This result reveals that after thinning down to ~10 μm, ceramic PZT can still maintain a much higher piezoelectric coefficient than sputtered or sol-gel PZT thin films, although it is lower than the value (*d*_31_ = 300 pC/N) given in the datasheet of the bulk ceramic PZT-5H wafer.

### Resonant frequencies

**Fig. 5:**
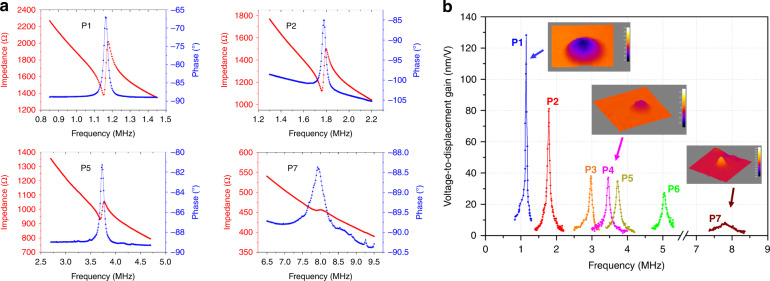
Measured resonant frequencies of the fabricated multi-frequency pMUT array. **a** Electrical impedances of different pMUT elements. **b** Mechanical vibration at the center of pMUT diaphragms in the frequency domain

The resonant frequencies of pMUTs were evaluated through electrical impedance measurements and mechanical vibration tests. The electrical impedance of pMUT elements (P1 to P7) in a multi-frequency pMUT array was measured with an impedance analyzer (4294 A, Agilent Inc., USA). Resonant frequencies of 1.15 MHz, 1.79 MHz, 2.97 MHz, 3.47 MHz, 3.73 MHz, 5.04 MHz, and 7.80 MHz are observed, corresponding to pMUT elements labeled P1 to P7, respectively. Although the resonant frequencies of the fabricated pMUTs are affected by the thickness variation of the CMP, the central part of the wafer has good thickness uniformity. Most of the working pMUTs are picked up from this region, whose measured resonant frequency variations are within 5% of the typical values. Figure 5a shows representative electrical impedance spectra measured from P1, P2, P5, and P7. Based on the measured resonant frequencies *f*_*r*_ and anti-resonant frequencies *f*_*a*_, the effective electromechanical coupling coefficient $k_{eff}^2$ of pMUTs can be calculated by:

1 $$k_{eff}^2 = 1 - \left( {\frac{{f_r}}{{f_a}}} \right)^2$$

The calculated $k_{eff}^2$ values are 5%, 4.4%, 4.2%, and 2.5% for P1, P2, P5, and P7, respectively. The obtained $k_{eff}^2$ values are relatively higher than those of pMUTs based on AlN and sol-gel PZT thin films^43,44^ but smaller than those of typical bulk PZT disks or plates^45^. The potential reason could be large parasitic capacitances generated between the bonding pads/top electrode lines and the unpatterned bottom electrode layer.

The mechanical vibration of the multi-frequency pMUT array was measured in air using a digital holographic microscope (DHM-R2100, LynceeTec., Switzerland). Each pMUT element was electrically driven at 2 V_pp_ with a sweeping frequency, and the time-domain vibration at the center of the diaphragm was measured and used to calculate the resonance spectrum in the frequency domain. The results are plotted in Fig. 5b and show the same resonant frequencies as those observed in the electrical impedance measurement. The corresponding voltage-to-displacement gains at resonance range from 8 nm/V to 130 nm/V for the pMUT elements. Some of the measured mode shapes are also shown in the figure.

### Ultrasound experiments

**Fig. 6:**
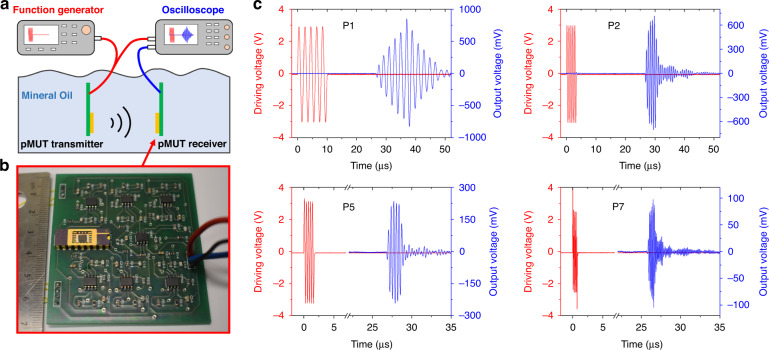
Acoustical characterization of the fabricated multi-frequency pMUT array. **a** Schematic of the experimental setup. **b** Photograph of a pMUT receiver on the PCB. **c** Ultrasound transmitting and receiving results for different pMUT elements at different frequencies

In PAI, photoacoustic signal detection relies on ultrasonic transducers in contact with biological tissues through a coupling medium, typically water or oil. Therefore, it is necessary to evaluate the acoustic sensing performance of the fabricated pMUT arrays at different frequencies in liquid. In this work, ultrasound transmitting and receiving experiments were conducted in mineral oil with two fabricated multi-frequency pMUT arrays. Figure 6a shows a schematic of the experimental setup. In the experiment, one fabricated pMUT array was used as the ultrasound transmitter, while the other was used as the receiver. To match the electrical impedance of the pMUTs and amplify their output signals, a seven-channel preamplifier was designed and fabricated on a printed circuit board (PCB). The preamplifier was designed to have two stages for a 30 dB gain, which consisted of a first-stage charge amplifier and a second-stage voltage amplifier^35^. The fabricated pMUT array was first wire bonded on a dual in-line package, which was then soldered on the PCB, as shown in Fig. 6b. For electrical insulation, a thin layer of Parylene C (~200 nm) was coated on the entire PCB, and experiments were conducted in mineral oil.

In the experiment, five sine bursts at specific frequencies were applied to drive specific pMUT elements to generate ultrasound signals, which propagated and were detected by the corresponding pMUT elements of the receiver placed at about 4 cm away. The output signal of the pMUT was synchronized with the driving signal and recorded with an oscilloscope. The experimental results show that the multi-frequency pMUT array can generate strong ultrasound signals at frequencies ranging from 300 kHz to 8 MHz and detect them. Figure 6c shows representative ultrasound signals generated and detected by P1, P2, P5, and P7 at frequencies of 500 kHz, 1.5 MHz, 3 MHz, and 7 MHz, respectively, where the time delay between driving signals and output signals corresponds to the distance between the pMUT transmitter and the receiver. The resonant frequencies of the pMUTs are smaller in oil than in air due to the added radiation mass. The ultrasound experiments verify the multiple working frequencies of the fabricated multi-frequency pMUT arrays in liquid and demonstrate their strong acoustic sensing performance at a large distance of 4 cm.

### Photoacoustic imaging experiments

**Fig. 7:**
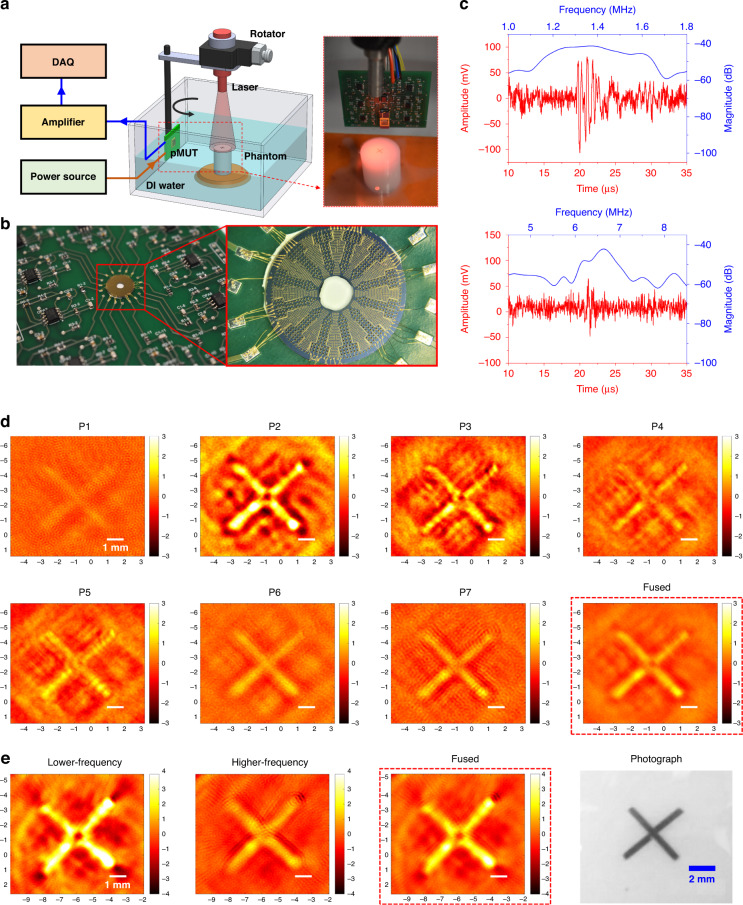
Photoacoustic imaging experiments. **a** Schematic of the experimental setup (inset: a photograph of the phantom). **b** Photograph of a dual-frequency pMUT array packaged on the PCB. **c** Photoacoustic signals detected by pMUT elements P2 (top) and P7 (bottom). **d** Reconstructed single and fused photoacoustic images obtained by the multi-frequency pMUT array. **e** Reconstructed single and fused photoacoustic images obtained by the dual-frequency pMUT array and a photograph of the imaging target.

To validate the PAI performance of the fabricated dual- and multi-frequency pMUT arrays, PAI experiments were conducted on a phantom made of an agar post (3 cm in diameter and 6 cm in height) with two 0.5 mm-diameter pencil leads embedded as the imaging target (a ‘+’ shaped pattern). The PAI system consists of a pulsed laser source, a mechanical scanning subsystem, and an acoustic signal detection and processing subsystem. Figure 7a shows a schematic of the experimental setup, where short laser pulses (720 nm wavelength, 6 ns duration, and 20 mJ energy) generated from an OPO laser source (Phocus Mobile, Opotek Inc., USA) are used to excite the target to generate photoacoustic waves. The pMUT array integrated on the PCB is controlled by a rotator to scan around the phantom at a step of 3° to detect photoacoustic signals. At each position, the pMUT elements detect a photoacoustic A-line signal after 40 times averaging, and a total of 120 A-line signals are acquired, for a full revolution in 5 minutes. The output signals from the pMUT are further amplified by a pulser/receiver (5077-PR, Olympus Inc., Japan) and then sent to a data acquisition (DAQ) board (PCI-5152, National Instruments Inc., USA). Photoacoustic images are reconstructed based on the acquired data using a delay and sum algorithm^46^.

In the experiment, the square-shaped multi-frequency pMUT array and the ring-shaped dual-frequency pMUT array were packaged and wire-bonded on 7-channel and 12-channel interface circuit boards, respectively. Although the dual-frequency pMUT array was designed with 60 lower-frequency channels and 60 higher-frequency channels, due to the limited space on the PCB and DAQ board, only 12 channels were included on the PCB. Figure 7b shows a photograph of a dual-frequency pMUT array packaged on the PCB. As shown in the figure, five lower-frequency pMUT channels along the radial direction were wire-bonded together on the PCB as one lower-frequency output channel. Similarly, five higher-frequency pMUT channels were connected together as one higher-frequency output channel. The photoacoustic signals acquired from one lower-frequency output channel and one higher-frequency output channel were used to reconstruct photoacoustic images.

Photoacoustic signals were successfully detected by pMUT elements with different resonant frequencies from dual- and multi-frequency pMUT arrays placed at distances of 3 cm to 5 cm away from the imaging target. Figure 7c shows two representative photoacoustic A-line signals detected by pMUT elements P2 and P7 from a multi-frequency pMUT array. By performing fast Fourier transform (FFT) of the detected photoacoustic A-line signals, the frequency response of the corresponding pMUT elements is obtained. As shown in the figure, the photoacoustic signals detected by the lower-frequency pMUTs (P2) and the higher-frequency pMUTs (P7) exhibit center frequencies of 1.37 MHz and 6.7 MHz, respectively. The signals detected by the lower-frequency pMUTs also display relatively larger amplitudes and longer durations.

Figure 7d shows the reconstructed photoacoustic images based on the photoacoustic signals detected by different pMUT elements from a multi-frequency pMUT array. In the current photoacoustic imaging system, the pMUTs are rotated around the central target only at a fixed height. Therefore, the acquired photoacoustic image is a 2D image showing the top view of the imaging target. Adding rotation at different heights is needed to reconstruct 3D images. It can be seen that all reconstructed images can clearly show the cross pattern of two pencil leads, which is displayed in a photograph of the actual imaging target in Fig. 7e. It is also obvious that the reconstructed photoacoustic images exhibit different resolutions and contrasts. As revealed by the images, the lower-frequency pMUTs (P1 to P4) can only delineate the major structures, with bright points in the images representing strong photoacoustic signals. In contrast, the images reconstructed from the signals detected by the higher-frequency pMUTs (P5 to P7) not only exhibit higher resolution but also provide better accuracy in terms of the target size. Moreover, the pMUT frequency should be selected according to the target size since the peak frequencies of the photoacoustic signals are inversely proportional to the absorber size. The image acquired by P1 has the lowest contrast because its working frequency is not high enough for imaging such small targets.

To better demonstrate the advantages of using multi-frequency pMUT arrays for PAI, a fused image was reconstructed based on all the above reconstructed photoacoustic images. Since the reconstructed photoacoustic images are 2D images with pixels exhibiting different intensities, an average intensity at each pixel can be obtained by summing the intensities at the same pixel of all the images reconstructed by the pMUT elements (P1 to P7) and dividing by the number of images. The fused photoacoustic image was reconstructed and is shown in Fig. 7d. Compared with the images reconstructed from single elements, the fused image exhibits higher resolution, better contrast, and more accurate target dimensions.

Reconstructed photoacoustic images based on the signals acquired from the lower-frequency pMUTs and higher-frequency pMUTs of a dual-frequency pMUT array are shown in Fig. 7e. Similar to the images obtained with P2 and P7 of the multi-frequency pMUT array, the cross pattern is observed in both images, but different resolutions and contrasts are exhibited. The image reconstructed by the lower-frequency pMUT elements reveals the cross pattern with a low resolution, while the image reconstructed by the higher-frequency pMUT elements shows the cross pattern more clearly, with the measured width closely matching the 0.5 mm diameter of the pencil lead used. Figure 7e also shows a fused image based on images reconstructed from both lower-frequency and higher-frequency pMUT elements, which has both high resolution and good contrast.

The PAI results demonstrate the capabilities and advantages of the fabricated dual- and multi-frequency pMUT arrays for PAI. Dual-frequency pMUT arrays can be considered as one of multi-frequency pMUT arrays. Each frequency component in a multi-frequency pMUT array covers a unique combination of imaging resolution and depth. Thus, the more frequency components a pMUT array has, the better the overall imaging resolution and depth it can provide. Compared with other works on multi-frequency pMUT arrays, this work presents a more comprehensive characterization of the multi-frequency performance of pMUT arrays and demonstrates PAI results with higher resolution and better contrast^47,48^. By using thin ceramic PZT, a 9-µm-thick piezoelectric film is obtained, with thickness more than four times greater than that of sol-gel PZT or AlN, which typically have thicknesses of <2 µm. Enabled by the increase in film thickness, a wider resonant frequency tuning range from 1 MHz to 8 MHz is achieved on pMUT elements with diameters tuned from approximately 86 µm to 300 µm.

However, due to the limitations of the current PAI setup, only 2D photoacoustic images can be reconstructed without depth scanning. In the future, by upgrading the experimental setup and designing a complicated phantom, 3D photoacoustic images can be obtained to quantify the imaging resolution and depth of the pMUT arrays. The employed sensing channels of the ring-shaped dual-frequency pMUT array in this study are also limited by the current size of the PCB and ASIC. By reducing the size of the multichannel interface circuit and expanding the number of ASIC channels to support the total 120 channels of the pMUT array, beam forming and fast imaging capabilities are expected, with photoacoustic signals from a 360° view acquired simultaneously.

## Conclusions

This work demonstrates the advantages and feasibility of fabricating dual- and multi-frequency pMUT arrays based on thin ceramic PZT for endoscopic PAI applications that can simultaneously achieve high spatial resolution and a large signal-to-noise ratio. A square-shaped multi-frequency pMUT array with 285 pMUT elements incorporating seven different resonant frequencies ranging from 1 MHz to 8 MHz and a ring-shaped dual-frequency pMUT array with 2520 pMUT elements were successfully developed based on a 9-µm-thick ceramic PZT layer. The dual- and multi-frequency performance of the fabricated devices was fully characterized via electrical impedance measurements, mechanical vibration tests, and ultrasound transmitting and receiving experiments, with all the pMUT elements working as expected.

Moreover, PAI experiments were conducted using the fabricated dual- and multi-frequency pMUT arrays, which successfully detected photoacoustic signals and reconstructed photoacoustic images of two pencil leads embedded in an agar phantom. Photoacoustic images reconstructed by pMUT elements with different frequencies clearly showed the relations between the imaging resolution and contrast, the center frequency of pMUTs, and the absorber size. Photoacoustic signals acquired from pMUTs with different frequencies were also combined to reconstruct fused images, which revealed the advantages of using dual- and multi-frequency pMUT arrays to provide comprehensive photoacoustic images.

In the future, with the miniaturization of the multichannel circuit board, the developed dual- and multi-frequency pMUT arrays are expected to be assembled into probes and applied in endoscopic PAI for imaging multi-size targets, obtaining both high resolution and large imaging depth.
